# Therapeutics of Epilepsy

**Published:** 1876-12

**Authors:** Allan McLane Hamilton

**Affiliations:** Visiting Physician to Epileptic and Paralytic Hospital, Blackwell’s Island, New York City, Etc.; 123 East 30th Street, New York


### EXAMINER.

Vol. XXXIII.—DECEMBER, 1876.—No. 12.

### Original Communications.

### THE THERAPEUTICS OF EPILEPSY.

By ALLAN McLANE HAMILTON, M.D.,

Visiting Physician to Epileptic and Paralytic Hospital, Blackwell's Island, New York City, Etc.

The object of this paper is the discussion of the present method of treating that most discouraging and imperfectly understood form of disease, Epilepsy. I wish more particularly to consider the value of the bromides, and at the same time to detail recent investigations undertaken to support a statement I made at the last meeting of the American Neurological Association, where I advocated the *medium dose*, and endeavored then to show that of late there is an unwise tendency to administer these drugs in dangerous quantities.

I may be pardoned, perhaps, in calling attention to certain practical points which may appear unimportant to some, but an experience gained from the management of a great many cases teaches me they are to be carefully considered in selecting a plan of treatment. These simple indications, I am convinced, are too often overlooked, even by painstaking and careful medical men. I allude to the necessity for discovering the exciting cause. I am every day made to feel that the idiopathic cases do not form so large a proportion as they were once thought to. With this belief I am satisfied that empiricism and routine management are bad methods. Any one who examines all his cases thoroughly will recognize the delicate shades in epilepsy, variations which are exhibited in other diseases presenting more pronounced and better defined symptoms; consequently there are evidences of pathological action, which are not always grouped alike, and consequently all cases are not to be treated in the same manner. I ascribe the moderate success I have had in the management of this disease to the recognition of these differences.

Not only may obstinate epilepsy result from masturbation, but it may be due to many of the diseases of women, and is produced by other eecentric irritations of various kinds, or by centric irritation, such as may be associated with toxæmia.

Sir Charles Locock (*Med. Times and Gazette*, May 23, 1853,) called attention to many cases that he had treated where uterine irritation was the exciting cause; and I think others have had the same experience. In one of Locock's cases the patient was affected particularly at the menstrual periods.

Some of these peripheral causes are curious in the extreme. Through the kindness of Dr. Gibney, of New York, I was enabled to see a child who had accidentally injured her ear with her parasol, the brass tip of which remained for some time imbeded in the external auditory meatus. As a result, convulsions of an epileptic character were caused, and it was not until some time afterward that the foreign body was discovered and removed. In another case I treated, the Epilepsy was unmistakably due to a bad habit the woman had of wearing a number of heavy garments about her hips, which produced some uterine change. When this condition of affairs was noticed, and the skirts removed, she immediately recovered. At the root of many epilepsies, as well as other neuroses, are reflex causes — the starting point being the organs of digestion, or those contained in the pelvis. Of course there are varieties of epilepsy of an idiopathic nature, or others caused by traumatism or organic disease; and these will defy the best directed efforts of the physicians, and we can do nothing.

We should not lose sight of syphilitic epilepsy where pain always precedes the attack. It is generally curable.

In prescribing for our patient there are five indications to observe:

1. Removal of exciting cause, if possible.
2. The diminution of exaggerated reflex susceptibility of the medulla.
3. Equalization of cranial circulation.
4. Abortion of paroxysms.
5. Improvement of general condition.

For the accomplishment of these, it is imperative that a judicious and discreet selection of drugs should be made; and as those which are the most effective I may mention:

The Bromides: Sodium, Potassium, Calcium, Lithium, Iron.
Belladonna.
Digitalis.
Strychnine.
Ergot.
Arsenic.
Amyl Nitrite.
Tri-Nitro-Glycerin.
Cod Liver Oil.

I have not classified these remedies, as it is unnecessary to do so; but will now say a word in regard to their usefulness:

No one drug can be declared a specific — as I am sorry to see has been done — and we must not be too eager to accept the sanguine results of certain over-enthusiastic authorities, and be governed thereby. I allude more especially to the almost universal use of the bromides, to the exclusion of everything else, and also to their employment in quantities, which often ruin the patients, and at any rate produce a condition of diminished vitality — which is inconsistent with any hope of success. [1] Radcliffe's idea in this respect is a good one: "There is reason to believe that the therapeutics of convulsion

[1] Radcliffe. Pain, Epilepsy and Paralysis, p. 215.

must be based upon the notion that vital power has to be reinforced, and not upon the contrary opinion." What the proper dose is, has not been clearly settled by any one. There are neurologists who believe in toxic doses, and there are others who prescribe quantities which are almost small enough to be inert. In England it has been the custom to prefer the very small doses. I have seen the prescription of a very distinguished general practitioner, who thinks five grains of the bromide of potassium a sufficient dose. [1] Ringer recommends from 30—60 grains in the day; [2] Radcliffe, 45 grains; [3] Russell Reynolds, 30—90 gains; [4] Bartholow, 30—240; and [5] Hammond, 90—240 grains during the day. [6] Handfield Jones remarks that there is a great difference in the tolerance of individuals in regard to the bromides—some persons not being able to stand five grains, while others will not be affected by doses of less than forty grains in amount.

My own experience has taught me that the best effect can be gained by the repeated administration of sixty grains in the twenty-four hours. The larger doses produce rapid bromism, while a medium dose seems to be better appropriated, but will do just as much mischief in the way of bromism as the larger ones, if given for a length of time. My records show me that the average time for development of symptoms of this kind is about three months, while anæsthesia of the fauces is produced in a few weeks, or even a much shorter time; and I agree with others that it is necessary to produce this condition before we can say that the medicine has produced its physiological effect. Brown-Séquard considers the appearance of acne to be an indication that the medicine has begun to do its work, in which opinion he is joined by [7] Dr. Putnam-Jacobi. [8] Voisin considers the "point of saturation to be indicated by the

[1] Handbook of Therapeutics, p. 92.
[2] Pain, Epilepsy and Paralysis, p. 202.
[3] System of Medicine, p. 323. Vol. II.
[4] Materia Medica and Therapeutics, p. 371.
[5] Clinical Lectures on Nervous Diseases.
[6] Functional Nervous Diseases, p. 325.
[7] Oral communication at Am. Neurological Association.
[8] Voisin, Archiv. de Médecine, Jan., 1873.

anæsthesia of the pharynx and nares, so that in one case nausea is not produced by titillation with a spoon, and in the other sneezing and weeping does not follow the introduction of a straw into the nasal cavity." I should consider the latter a rather severe test. According to Danton[1] the bromides act as vascular medicaments, diminishing excito-motor power. They act on the unstriped muscular fiber, producing local anæmia and moderating excitation resulting from temporary or permanent congestion. [2] "They are agents that pass very rapidly into the blood (Ringer), and consequently their effects are very immediate, and they accumulate till the point of saturation is reached before they are eliminated in anything like considerable amounts." We are all aware that repeated and large doses of these drugs are followed by a most disagreeable and pernicious state of affairs. [3] Voisin has referred to two forms of poisoning, which he has divided into the slow and rapid. In the first the complexion becomes muddy, the eyes sunken, sight and hearing poor, and memory obscure. The patient cannot write, and cannot express himself, as he forgets words — there is tremulousness. In the other variety of the *slow* form there is dementia, or delirium with maniacal outbursts. Ataxia is also a feature of this variety. In the *rapid* form — that with which we are most familiar — somnolence, headache, uncertain walk, difficulty of speech, loss of expression, "fishiness" of the eyes, drooling of saliva, etc., etc., are the ordinary symptoms.

Various grades of toxæmia, or even a state which Voisin calls the "cachexie bromique," and which terminates in a typhoid condition, may result from a reckless use of this drug.

As regards the variety of bromide, I think the sodic is the most reliable and stable; the potassic salt varying very much in strength. The others either have a tendency to deliquesce, or are expensive. It will be advisable to keep the solution in a tight-stoppered bottle, and have fresh quantities put up constantly, as it is very apt to undergo changes — in which

[1] Danton. Thèse de Paris, 1874.
[2] Ringer. Handbook of Therapeutics, p. 91.
[3] Voison. Archiv. de Médecine, Jan., 1873.

the bromine is evolved. And now a word regarding the time of administration. It has been shown repeatedly that these salts are much better absorbed when the stomach is empty. I have found also that a heavy dose at night is apt to do more good than if the amount prescribed is equally divided up through the day. In a great many patients I have found the attacks to occur at the waking hour, and I suppose this is due to the sudden change in the cerebral circulation. A mild diffusive stimulant has overcome this, and in many cases warded off the attack. I direct my patients who have their convulsion at this time to keep a glass of sherry or a small quantity of Spts. Ammoniæ Aromaticus near at hand, to be taken before arising. Cold douches to the head are valuable. If the attacks be irregular, it will be found necessary to divide up the dose.

The treatment of the disease in women should be directed as well to the pelvic organs. It will be found that the bromides will markedly affect the flow, and relieve the pain or uneasiness which is connected with the menstrual period. Locally I have found that cold applied for a few minutes daily over the ovaries, will modify the attacks should they be connected with irritation of any of the pelvic viscera. The progress of the disease should be soon modified by the doses I have recommended, and it will be seen by the table condensed from that prepared by Dr. Hollis and published in the *British Medical Journal*,[1] that even smaller doses modified or cured the majority of the cases he cites. At the Epileptic and Paralytic Hospital, where most of the cases are the very worst that can be collected as regards chronicity, I find that sixty grains a day will cut short the attacks of a great many patients, and I have cured a number of private patients by this method. Dr. Hollis' cases were not selected, and are evidently hospital patients, like my own.

[1] Br. Med. Journal, July 1, 1876, p. 4.

### ANALYSIS OF ELEVEN CASES OF EPILEPSY.

S. B.—Sodic Bromide. P. B.—Potassic Bromide.

| NO. OF CASE. | SEX & AGE. | DURATION OF DISEASE. | AVERAGE NO. OF ATTACKS BEFORE TREATMENT | MAXIMUM DOSE. | MINIMUM DOSE. | DIMINUTION. | REMARKS. |
|---|---|---|---|---|---|---|---|
| 1 | Male, 15 | Since birth | 1–2 weekly. | S.B. gr. xx, t.i.d. | S.B. gr. xv, t.i.d. | 2 in 8 weeks | Weak intellect. |
| 2 | Male, 22 | Two years. | 1–2 weekly. | S.B. gr. xv. | ........ | 1 in 20 wks. | Disease followed sunstroke; treatment lasted 3 months. |
| 3 | Male, 25 | One year .. | 1 or more in week, sometimes many in a day ....... | S. B. xxv., P.B. gr. xxx | S.B. gr. ij.. | None in 8 weeks .... | Hard drinker, feeble intellect; Potassium salt inert. |
| 4 | Fem., 2 | 18 months . | 1–2 weekly, sometimes 3 in a day. | Very small doses..... | ........ | None in 8 weeks .... | Fits followed dentition; ricketty constitution. |
| 5 | Fem., 18 | One year .. | 1 a week... | S.B. gr. xxx | Gr. xx ..... | None in 4 weeks .... | Tuberculous disease. |
| 6 | Male, 18 | Five years. | 4 in week.. | S.B. gr. xv. | ........ | None from 5 weeks... | No affection of intellect. |
| 7 | Fem., 11 | Five years. | 2–3 in week | S.B. gr. xx. | S.B. gr. xv. | 1 in 5 weeks | Followed a blow; subject to headache. |
| 8 | Fem., 17 | Several mos | Sometimes 4–5 daily.. | S.B. gr. xv. | ........ | None after treatment. | Has bitten tongue. |
| 9 | Male, 20 | 19 years.... | 2–3 weekly. | S.B. gr. xl. | S.B. gr. xv. | No fits for 2 weeks... | No aura. |
| 10 | Male, 13 | Two years. | 3 weekly... | S.B. gr. xxv | S.B. gr. xv. | 1 in 3 weeks | Well developed disease, facies Epileptica well marked. |
| 11 | Male, 25 | Eleven yrs. | 1 in 2 weeks | S.B. gr. xx. | ........ | 1 in 5 weeks | No fits since beginning of treatment. |

By this table it will be seen that from fifteen to twenty grains of the sodic salt were required to immediately decrease the number of attacks.

Below will be found two tables. In one are tabulated the interesting features of twelve cases of epilepsy. They are old hospital patients, and had applied for admission after outside treatment had been exhausted. Even here the bromides, in the doses I have given, seem to do much for the sufferer. Syphilis, traumatism and actual insanity make the prognosis as bad as it well can be, and treatment is simply palliative. Large doses have aggravated many of these cases.

The other observations are selected from my note book, and are illustrative of the efficacy of the dose I have advocated.

Bromism occurred in spite of all I could do in most of them, though it was a mild form and under control. They were all patients of the better class, and of course had all the advantages of comfortable homes, attentive friends, substantial food and good air, although many of them were inclined to overeating, as in fact all epileptics are. In this respect there is an advantage in favor of the poorer patients, who cannot obtain rich food.

### ANALYSIS OF TWELVE CASES OF CHRONIC EPILEPSY.

S. BR.—SODIC BROMIDE. P. BR.—POTASSIC BROMIDE. H. BR.—HYDROBROMIC ACID.

| NO. | AGE. | DURATION OF DISEASE. | AVERAGE NO. OF ATTACKS BEFORE TREATMENT. | AVERAGE NO. OF ATTACKS DURING TREATMENT. | MAXIMUM DOSE OF DRUG. | MINIMUM DOSE OF DRUG. | FORMER TREATMENT. | DURATION OF PRESENT TREATMENT. | REMARKS. |
|---|---|---|---|---|---|---|---|---|---|
| 1 | 28 | 16 years | 1 monthly. | 1 in 2 months | P. Br. gr. xx., t.i.d. | P. Br. xv. t.i.d. | ............ | 6 months. | Has taken H. Br. for 1 month—fits increased. |
| 2 | 25 | 12 years | 6 monthly. | 1 monthly ... | H. Br. 1 dr., t.i.d... | ................ | S. Br....... | 1 month.. | Melancholic. |
| 3 | 25 | 20 years | 18 monthly. | 3 weekly...... | Ergot (for 1 month) | ................ | ............ | 6 months. | When under S. Br. attacks were 3 in month. |
| 4 | 27 | 14 years | 4 monthly. | 1 monthty ... | P. Br. gr. xx., t.i.d. | P. Br. xv. t.i.d. | S. Br....... | 1 month.. | Masturbates. |
| 5 | 19 | 6 years | 7 monthly. | 1 monthly ... | S. Br. gr. xx., t.i.d. | ................ | P. Br....... | 4 months. | Hysterical. |
| 6 | 20 | 19 years | 2 monthly. | 1 in 6 weeks . | P. Br. gr. lx., daily. | S. Br. x. t.i.d .. | Chloral,etc. | 6 months. | |
| 7 | 22 | 10 years | 21 monthly. | 2 weekly...... | P. Br. gr. lx., daily. | ................ | ............ | 9 months. | Menstrual complication. |
| 8 | 22 | 6 years | 7 monthly. | 1 weekly...... | P. Br. gr. xv., t.i.d. | ................ | ............ | 6 months. | |
| 9 | 18 | 4 years | 8 monthly. | 1 monthly ... | F.ExErg.½ dr.,t.i.d | ................ | ............ | 4 months. | Bad habits. |
| 10 | 23 | 23 years | 3 monthly. | 1 in 2 months | P. Br. gr. xv,, t.i.d. | ................ | ............ | 4 months. | |
| 11 | 29 | 27 years | 5 monthly. | 1 in 3 months | S. Br. xx., t.i.d..... | ................ | ............ | 6 months. | Bad habits. |
| 12 | 25 | 12 years | 6 monthly. | 1 weekly...... | P. Br. gr. xx., t.i.d. | S. Br. xv. t.i.d.. | ............ | 3 months. | |

ANALYSIS OF FOUR SUCCESSFUL CURES OF EPILEPSY WITH MODERATE DOSES OF THE SODIC BROMIDE.

G. M.—GRAND MAL. P. M.—PETIT MAL.

| NO. | AGE. | SEX. | DURATION. | NO. ATTACKS BEFORE TREATMENT. | NO. OF ATTACKS DURING TREATMENT. | AFTER FIRST YEAR. | AVERAGE DOSE, ETC. | DURATION OF ACTIVE TREATMENT. | CONTINU'D SUBSEQUENT TREATMENT. | VARIETY OF DISEASE. | PERIOD UNDER OBSERVATION. | REMARKS. |
|---|---|---|---|---|---|---|---|---|---|---|---|---|
| 1 | 15 | M.. | 10 years | G. m., about 4 weekly; p.m. 10, 40 daily... | For first 3 mos. 5; for next 6 mos., none .. | 2 in 2 years. | 20 grains Sodic Brom., t.i.d.; auxil'ry treatment; Ergot, Arsenic, Iron, etc. ......... | About 9 mo. | X-xx grs. Sodic Bromide at night; strict hygienic care | gm. at night | 4 years..... | Bright boy; Epileptic habit almost confirmed; intelligent parents, who aided in general management & discipline; apparently idiopathic origin. |
| 2 | 29 | F .. | 12 years | 1 to 2 weekly; g. m.......... | After xx. grs., S. Br. 2 in a week, 3 in next 2 weeks, 1 in next 5 weeks, 2 in next month, 1 in next 4 mos. 1 in next 9 months ..... | 3 in 2 years. | Cod Liver Oil, Sodic Bromide, xxv. grs., t.i.d.; atropia hypoder'cally | About 19mo | Gr. x., t.i.d. ... | Mixed ..... | About 4 yrs | Over study; hysterical; no uterine trouble. |
| 3 | 36 | M.. | 9 years | 1 or 2 every 2 months; g.m. | S. Br. gr. xx. at night; 3 in 7 months ...... | 1 in 1½ yrs. | S. Br. gr. xx., at night ........ | About 2 yrs | Gr. x-xx., t.i.d. Sea voyage; cod liver oil. | Nocturnal. | About 3 yrs | Business man; overwork; supposed blow on head; attacks excited by over-eating. |
| 4 | 17 | F .. | 5 years | About 2 m'thly | S. Br. gr. xx., t. i.d.; Ergot increased them to about 5 m'thly; worse at menstrual periods; stopped Ergot; fits about once a month ...... | 1 in 2d year; none to date, Sep. 20, 1876. | S. Br. gr. xx., t. i.d.; uterine treatm't; Syr. Lacto-phos., Arsenic...... | About 4 mo. | Lacto-phos. of Lime; S. Br. gr. v., t.i.d. for 6 months. | Mixed; generally ma-tutional.. | About 4 yrs | Case undoubtedly dependent upon anteversion of uterus; immediate cessation of disease when the position of this organ was rectified. |

And now regarding the large doses. If the idea is to thoroughly ruin the patient's health, enfeeble his mind, or perhaps drive him to an asylum, the toxic administration may be indulged in. It is very true that sometimes a rapid restoration may be brought about by "Iron and Quinine;" but there are many cases where the recovery is not quite so complete as one could wish for. Memory is enfeebled, and there is a cachexia which remains for an indefinite time. A darker side of the picture is not always displayed when brilliant results are detailed. This is the list of demented and those that have died. My friend, Dr. Janeway, was present at the autopsies of two patients who died brominized—for certainly the examinations disclosed no other cause for death. I myself have seen several demented cases, and I have no doubt others could tell the same story.

Belladonna and its alkaloids are of great value when the seizures occur in the daytime, or are of the variety known as *petit mal.* I have injected the Sulphate of Atropia in $\frac{1}{64}$ gr. doses beneath the skin at the back of the neck with good effect, and have given it in the manner directed by Trousseau. In either way it should be administered until dryness of the throat is obtained, and should be given a patient trial. The property possessed by belladonna of blunting reflex susceptibility assures it a great advantage over other methods of treatment, when there are centers of irritation such as in gastric epilepsy.

In Ergot we have a remedy which controls the cranial circulation much more readily than any drug I am acquainted with. As the object is to diminish the congestion at the floor of the fourth ventricle, its combination with the bromides greatly increases the action of the latter. Ergotin may be given alone in the form of Bonjean's capsules.

To [1] Tyrrell belongs the credit of suggesting Strychnine. He believes that this remedy controls excitation of the medulla oblongata. In one individual who averaged fifty-one attacks in a month, the number was reduced by the Strychnine to

[1] Med. Times and Gazette, May and Aug., 1867.

eleven in two years. [1] Handfield Jones does not favor the remedy, nor do others, although it has advocates in this country. In small doses it certainly does good; but I have found that in larger doses than $\frac{1}{32}$ gr., ter in die, it rather aggravates the disease.

Arsenic is excellent, both for its anti-periodic and alterative action, and as an agent to relieve the acne. [2] Clemens, of Frankfort, has lately advocated the Bromide of Arsenic, but in such small doses as to seem useless. He claims for it remarkable virtue when the disease depends upon idiocy, and appears in patients with deformity of the skull. He reports two cures.

Where there is an irregularity of heart action, sluggish circulation, blueness or duskiness of the skin, I think digitalis is indicated; in fact, I generally use it in every chronic case. It is a drug well tolerated by epileptics, who can take it in surprisingly large doses.

An agent has been lately given to the profession which seemed all that was needed at first, but which I am convinced is very much over-estimated, except as an abortant. I speak of the Amyl Nitrite. Drs. Weir Mitchell, Zeigler and Alexander McBride, as well as several foreign writers, have praised it, and several cures have been reported. In epilepsy there seems to be a "habit," (if I may use the expression,) or tendency to periodicity. Amyl is well adapted to stop this, as is any other remedy of the same class. Crichton Browne alludes to the effects of this drug upon the *status epilepticus.* His patient had had a great succession of fits, and was at the point of death — the pupils were contracted to an intense degree, pulse 116, temperature 102°, with stertorous breathing. Voluntary movements and yawning were caused by inhalation of the Amyl Nitrite, and the patient subsequently raised his head and looked about him. Dr. Browne relates ten other cases which were seen with Dr. Mierson.

[1] Handfield Jones' Functional Nervous Affections, p. 326.

[2] Allg. Medicinische Central Zeitung, May 24, 1876.

Dr. C. Steketec [1] draws the following conclusion in regard to the action of this drug in Epilepsy:

"It exerts an important influence where the epilepsy is due to, or connected with cerebral anæmia, for the reason that it 'anticipates the attack when there are prodromata — cuts off the attack when it appears, relieves symptoms due to interrupted innervation after the attack—and the attacks become less frequent'" (? by the author). He also considers it injurious where the attacks are due to cerebral hyperæmia, for the reason that they last longer and become more frequent, and when either maniacal or convulsive, increase in intensity.

My own experience with Amyl Nitrite has clearly settled in my mind the fact that it has great virtues in cutting short or averting attacks, but that it has no permanent influence. Whether we can or cannot make the delicate distinctions of Dr. Steketec, future clinical experience I think must decide. Those who have used it say that it does good in a very limited number of cases; and it is a difficult task to decide which are to be benefitted. I have tried it in every grade of Epilepsy, and find in some of the worst cases, where the fits occur all through the day, with very slight intervals, and even where there is time enough to be prepared, that it is often of no avail. It may be given enclosed in the little glass capsules invented by Dr. McBride, of New York; for hospital use, and for patients who are not intelligent, in alcoholic solution.

I may be pardoned for bringing another remedy to the notice of the profession, and one that has never been used for this purpose. I allude to Tri-Nitro-Glycerine. Its properties are almost enough to intimidate the patient, but it is as powerful a medicinal agent as it is an explosive. The tenth part of a drop touched to the tongue is sufficient in a space of time which is almost inappreciable to produce a rapid cerebral hyperæmia. The face is flushed, the eyes become bright, and the temporal vessels throb, while at the same time there are the marked sensations of fullness. It produces more lasting congestion than does Amyl Nitrite, is much safer, and I have

[1] Thesis abstracted by Chicago Journal of Nervous and Mental Disease, April, 1874, p. 260.

found it to act better as an abortant than the latter. Any good pharmacist can prepare a solution containing one drop to ten of alcohol. This can be further diluted so that ten drops of alcohol shall contain one-tenth of a drop of the Nitro-Glycerine. It may be kept safely in this way, for alcohol prevents its explosion. A dose of a tenth of a drop is sufficient in the majority of cases.

Last of all, it seems almost unnecessary for me to direct attention to that most familiar remedy, Cod Liver Oil, which is so valuable in all nervous diseases.

Anstie treated a number of cases by Cod Liver Oil alone and cured seven out of twenty patients put upon this plan of treatment alone. Picrotoxin, a remedy recently brought forward, I have tried, and consider valuless.

The question of diet and personal habits are very important ones — particularly as the stomach is so often the seat of irritations which are transmitted to the over-active centers. Beyond the question of over-eating, it has been found that a vegetable diet is better suited to this class of patients. Mierson, in the last volume of the West Riding Reports, publishes cases and makes comparisons between those epileptics placed upon a meat and vegetable diet. The results pointed to the superiority of the latter. As the greater number of epileptics have inordinate appetites, the diet should be strictly regulated.

It is a good plan, I think, to combine the remedies I have alluded to; and in conclusion I take the liberty of presenting a prescription I have used for several years:

| | | |
|---|---|---|
| ℞ | Strychniæ Sulph. .......................... | gr. j. |
| | Fl. Ext. Ergotæ .......................... | ℥ iss. |
| | Sol. Potass. Arsenit. .......................... | ʒ ij. |
| | Sodii Bromidi .......................... | ℥ iss. |
| | Tr. Digitalis .......................... | ʒ iij. |
| | Aquæ Menth. pip. .......................... | ad. ℥ iv. |

M. Sig.: A teaspoonful before eating, in a half tumblerful of water.

If the attacks be of the form known as *petit mal*, I think either Ergot or Belladonna are our best agents. With either form of treatment it may be found often necessary to use auxillary general treatment. The syrup of the combined phosphates, or the syrup of the Lacto-Phosphate of Lime, are good adjuncts; and salt baths, cold head douches, regular food, early hours, and the breaking off of bad habits, will often cure the disease, even when it has lasted for many years.

As a last resort, should continued medication prove useless, the actual cautery or a deep seton at the back of the neck will occasionally arrest these bad cases.

123 East 30th Street, New York, Sept. 27, 1876.

